# Awareness of Marketing of Heated Tobacco Products and Cigarettes and Support for Tobacco Marketing Restrictions in Japan: Findings from the 2018 International Tobacco Control (ITC) Japan Survey

**DOI:** 10.3390/ijerph17228418

**Published:** 2020-11-13

**Authors:** Lorraine V. Craig, Itsuro Yoshimi, Geoffrey T. Fong, Gang Meng, Mi Yan, Yumiko Mochizuki, Takahiro Tabuchi, James F. Thrasher, Steve S. Xu, Anne C. K. Quah, Janine Ouimet, Genevieve Sansone, Janet Chung-Hall

**Affiliations:** 1 Department of Psychology, University of Waterloo, 200 University Ave. W., Waterloo, ON N2L 3G1, Canada; gfong@uwaterloo.ca (G.T.F.); gmeng@uwaterloo.ca (G.M.); mi.yan@uwaterloo.ca (M.Y.); s4xu@uwaterloo.ca (S.S.X.); ackquah@uwaterloo.ca (A.C.K.Q.); j2ouimet@uwaterloo.ca (J.O.); gsansone@uwaterloo.ca (G.S.); jchunghall@uwaterloo.ca (J.C.-H.); 2 Division of Tobacco Control Policy Research, National Cancer Center Japan, 5-1-1 Tsukiji, Chuo-ku, Tokyo 104-0045, Japan; iyoshimi@ncc.go.jp; 3 Ontario Institute for Cancer Research, 61 University Ave., Suite 510, Toronto, ON M5G 0A3, Canada; 4 School of Public Health and Health Systems, University of Waterloo, 200 University Ave. W., Waterloo, ON N2L 3G1, Canada; 5 Japan Cancer Society, 13th Floor, Yurakucho Center Bldg. 2-5-1, Yurakucho, Chiyoda-ku, Tokyo 100-0006, Japan; yumiko.mochizuki@gmail.com; 6 Cancer Control Center, Osaka International Cancer Institute, Chome-1-69 Otemae, Chuo Ward, Osaka 541-8567, Japan; tabuchitak@gmail.com; 7 Department of Health Promotion, Education & Behavior, Arnold School of Public Health, University of South Carolina, Columbia, SC 29208, USA; thrasher@mailbox.sc.edu; 8 Tobacco Research Department, Center for Population Health Research, National Institute of Public Health, Cuernavaca, Morelos 62100, Mexico

**Keywords:** Japan, marketing, heated tobacco products, cigarettes

## Abstract

Japan is one of the world’s largest cigarette markets and the top heated tobacco product (HTP) market. No forms of tobacco advertising, promotion, and sponsorship (TAPS) are banned under national law, although the industry has some voluntary TAPS restrictions. This study examines Japanese tobacco users’ self-reported exposure to cigarette and HTP marketing through eight channels, as well as their support for TAPS bans. Data are from the 2018 ITC Japan Survey, a cohort survey of adult exclusive cigarette smokers (*n* = 3288), exclusive HTP users (*n* = 164), HTP-cigarette dual users (*n* = 549), and non-users (*n* = 614). Measures of overall average exposure to the eight channels of cigarette and HTP advertising were constructed to examine differences in exposure across user groups and products. Dual users reported the highest exposure to cigarette and HTP advertising. Tobacco users (those who used cigarettes, HTPs, or both) reported higher average exposure to HTP compared to cigarette advertising, however non-users reported higher average exposure to cigarette compared to HTP advertising. Retail stores where tobacco or HTPs are sold were the most prevalent channel for HTP and cigarette advertising, reported by 30–43% of non-users to 66–71% of dual users. Non-users reported similar exposure to cigarette advertising via television and newspapers/magazines as cigarette smokers and dual users; however, advertising via websites/social media was lower among non-users and HTP users than among cigarette smokers and dual users (*p* < 0.05). Most respondents supported a ban on cigarette (54%) and HTP (60%) product displays in stores, and cigarette advertising in stores (58%).

## 1. Introduction

Japan is the fifth largest cigarette market in the world and the largest market for heated tobacco products (HTPs). In 2018, adult smoking prevalence in Japan was 17.9% (27.8% males, 8.7% females). In 2018, there were about 19 million smokers and 133 billion cigarettes were sold [1,2]. However, the volume of cigarette sales declined by 32% or 62.6 billion cigarettes between 2011 and 2018. This decrease was slow and steady between 2011 and 2015, with an accelerated decline beginning in 2016 as heated tobacco products (HTPs) became established in the marketplace [3]. In Japan, the IQOS HTP device was first introduced by Philip Morris International (PMI) in 2014, followed by the launch of Ploom TECH by Japan Tobacco Inc. (JT) in March 2016, and glo by British American Tobacco (BAT) in December 2016. Prevalence of HTP use in Japan has risen rapidly from 0.2% in 2015 to 11.3% in 2019, especially among smokers, males, and young adults [4]. Another indication of the growing popularity of HTPs is its rising market share, reaching 21.7% in total tobacco sales volume by September 2018 [5].

HTPs are a new generation of electronic nicotine delivery devices that heat tobacco to a temperature high enough to produce an inhalable aerosol, but the temperature is below that which is required for full combustion [6]. Although there is some evidence that HTPs may involve some partial combustion [7], the tobacco industry argues that because HTPs do not involve combustion, they are potentially less harmful than combustible cigarettes [8,9,10]. While several independent studies have reported significantly lower emissions of some chemicals/toxicants from HTPs, it is not clear whether reduced exposure means reduced risk to HTP users [7,11,12,13,14].

Since the introduction of HTPs in Japan, tobacco companies have used intensive marketing and promotion strategies to increase product sales. For example, industry websites market HTPs as a reduced-risk product and a “less harmful” and “clean” alternative to combustible cigarettes with messages that these products “are free from fire, ash and smoke”, “without the cigarette-like smell”, and offer “a 99% reduction in the constituents recommended by WHO for reduction in cigarette smoke” [10,15]. HTPs are sold and marketed to consumers in IQOS “boutique” stores nationwide, retail establishments, and e-commerce websites and are marketed in traditional media channels such as TV, newspapers, posters, and billboards, as well as non-traditional channels such digital marketing through social media and internet platforms [6,16]. IQOS packaging is described as having youth-appealing qualities “resembling iPhones and other high-end smartphones” and product flagship stores bear a similarity to high-end technology brand stores [17]. The industry also uses packaging design, incorporating distinctive colors and images, to promote HTP tobacco sticks that are branded with established names such as Marlboro, Mevius, or Kent. Growth of the Japanese HTP market is attributed to consumer interest in innovative technology, the de facto ban on electronic cigarettes, and weak restrictions on tobacco advertising and promotion [6].

As a Party to the WHO Framework Convention on Tobacco Control (FCTC) since 2005, Japan is obligated under Article 13 to implement a comprehensive ban on direct and indirect forms of tobacco advertising, promotion, and sponsorship (TAPS). However, there are currently no laws prohibiting TAPS for either cigarettes or HTPs in Japan. Restrictions on TAPS operate as a form of “industry self-regulation” under the Tobacco Business Act (TBA) (1984), which is administered by the Ministry of Finance. Article 40 of the TBA calls on advertisers to make “efforts that their advertising not be excessive” [18]. Non-binding guidelines issued by the Ministry of Finance in 2004 refer to Japan’s commitment to the FCTC and the need to prevent minors from smoking when considering the location and content of advertising [19]. The guidelines discourage advertising on TV, radio, internet, newspapers, magazines, and other publications except when it is technologically possible to limit the target audience to adults. Advertising is permitted on posters, billboards, buildings or other structures that are tobacco points of sale or areas designated for smoking, but is restricted in “highly public places”. Distribution of tobacco samples, flyers, catalogues, pamphlets, etc. and sales promotion products to adults are permitted, but not in “highly public places” [19]. Industry sponsorship of events is permitted if participants, organizers, and the target audience are adults. The guidelines do not apply to corporate advertising, advertising designed to promote good smoking etiquette or to prevent minors from smoking or other activities that do not promote smoking [19]. 

It is well-documented that tobacco marketing promotes tobacco consumption. Numerous studies have found a dose-response relationship between tobacco advertising and uptake and progression to regular tobacco use among young people [20,21,22]. Until recently, most previous research has focused on the association between cigarette advertising and smoking behaviors. With the growing popularity of alternative nicotine delivery products, recent studies have examined exposure to advertising of vaping products among adults and youth. Surveys in Canada, the US, England, and Australia show that self-reported exposure to marketing of vaping products generally reflects country and channel-specific advertising bans, with the exception of online channels which are difficult to regulate and enforce [23,24,25,26]. Youth smokers and/or vapers were more likely to report advertising exposure compared to non-smokers and non-vapers. Youth who smoke and vape were more likely to report exposure to vaping product ads through all 15 channels of exposure compared to never users, while exclusive smokers and vapers were more likely to report exposure through most channels (e.g., stores that sell cigarettes, websites or social media) [24]. Adults who smoke cigarettes and vape reported higher exposure to both cigarette and vaping product advertising through any channel compared to exclusive smokers and vapers. Studies show that exposure to marketing of vaping products is associated with lower harm perceptions, greater intentions to use vaping products, and higher rates of trial among youth and young adults [27,28,29,30].

Few studies have examined levels and correlates of exposure to HTP marketing. In April 2019, the Food and Drug Administration (FDA) authorized the marketing of IQOS via the premarket tobacco product application (PMTA) pathway. In May 2020, FDA granted limited authorization to market IQOS as a Modified Risk Tobacco Product (MRTP) [31], allowing claims that IQOS reduces exposure to harmful chemicals, but not allowing claims that IQOS reduces risk/harm. This distinction between reduced exposure and reduced harm is an important one, although it is not clear whether consumers can make that distinction. A review of qualitative and quantitative studies conducted by PMI found that consumers perceived reduced exposure claims from IQOS marketing materials (such as brochures, packs, and direct mail) as reduced risk claims [12]. A study of marketing and perceptions of IQOS in Japan and Switzerland suggested that consumer reception to IQOS may differ by culture. Marketing IQOS as clean, chic and pure resonates well in Japan given the strong cultural values of order, cleanliness, quality and respect for others [32]. A study of Japanese smokers found that those who reported exposure to HTP advertising via various marketing platforms, including TV, billboards, social media, newspapers, magazines, bars/pubs, and stores where tobacco and HTPs are sold, were more likely to perceive HTPs as less harmful than cigarettes after controlling for HTP use [33].

The present study is the first population-level examination of exposure to HTP and cigarette advertising in Japan. The objectives were to: (a) examine differences in exposure to HTP and cigarette marketing across eight advertising channels among cigarette smokers, HTP users, HTP-cigarette dual users, and non-users; (b) compare overall exposure to HTP advertising vs. cigarette advertising across the four user groups; and (c) compare levels of support for policies to curb tobacco and HTP marketing.

## 2. Materials and Methods 

### 2.1. Data Source 

Data are from the International Tobacco Control (ITC) Japan Wave 1 (2018) Survey, a web-based survey of adult cigarette smokers (age ≥ 20 years), HTP users, HTP-cigarette dual users, and non-users (total *n* = 4615) conducted between February and March 2018.

A full description of the study methods (compliant with the Checklist for Reporting Results of Internet E-Surveys [34]) and survey design is available elsewhere [35,36]. Briefly, respondents were recruited from the Rakuten Insight panel in Japan. The Rakuten web panel is designed to be nationally representative of the Japanese population. Sampling weights were computed for all respondents and calibrated to target figures from the 2017 Japan Society and New Tobacco Internet Survey [37] to ensure that the final sample was representative of Japanese smokers, HTP only users, HTP-cigarette dual users, and non-users. The survey cooperation and response rates were 96.3% and 45.1%, respectively, which are high within the typical range for online surveys [38,39].

Study procedures and materials were reviewed and cleared by a University of Waterloo Research Ethics Committee (ORE#22508/31428, Waterloo, Canada). All participants provided informed consent. 

### 2.2. Measures

#### 2.2.1. HTP and Cigarette Advertising Exposure

Exposure to HTP (including products like IQOS, Ploom TECH, and glo) and cigarette advertising was assessed by asking respondents whether, in the prior six months, they had noticed advertisements for HTPs and for cigarettes through television, radio, newspapers or magazines, posters or billboards, store windows or inside stores where tobacco is sold (where tobacco and HTPs are sold for HTP advertising), email or text messages, websites or social media sites, and bars and pubs. Response options were: “yes/no/don’t use/don’t encounter/refused/don’t know”. Responses were dichotomized as “yes” or “no or don’t encounter”. Responses of “refused” and “don’t know” were coded as missing. An index on the number of channels exposed was also created for HTP and cigarette advertisements respectively by summing the “yes” responses from each of the eight channels (range: 0–8).

#### 2.2.2. Support for HTP and Cigarette Advertising Restrictions

Respondents were asked whether they support (a) a ban on cigarette displays inside shops/stores; (b) a ban on HTP displays inside shops/stores; and (c) a ban on cigarette advertisements inside shops/stores. Response options were “not at all/somewhat/a lot/refused/don’t know”. Responses were dichotomized to indicate support (“somewhat/a lot”) or not (“not at all/don’t know”). Responses of “refused” were coded as missing.

#### 2.2.3. Covariates

Sample characteristics included sex (female, male), age (20–29, 30–39, 40–59, 60+), education (low: junior high school/high school, moderate: vocational school/junior college/technical college, high: undergraduate/postgraduate, no answer), and annual household income (low: <4 million yen, moderate: 4 to 8 million yen, high: >8 million yen, no answer).

#### 2.2.4. User Groups

Respondents were categorized as exclusive HTP users (defined as those currently who used HTPs at least weekly and smoked cigarettes “less than monthly” or “not at all”, *n* = 164), exclusive cigarette smokers (had smoked at least 100 cigarettes during lifetime, currently smoked cigarettes “at least monthly”, and used HTPs “less than weekly” or “not at all”, *n* = 3288), HTP-cigarette dual users (currently used HTPs “at least weekly” and smoked cigarettes “at least monthly”, *n* = 549); and non-users of cigarettes and HTPs (those who smoked cigarettes “less than monthly” or “not at all” and used HTPs “less than weekly” or “not at all”, *n* = 614). 

### 2.3. Data Analysis

Survey logistic regression models were estimated to examine differences between the four user groups (exclusive HTP users (herein called “HTP users”, exclusive cigarette smokers (herein called “smokers), HTP-cigarette dual users (herein called “dual users”), and non-users) on their exposure to the eight cigarette and HTP advertising channels and support for TAPS bans. The average number of channels exposed to among the eight advertising channels common to cigarette and HTPs was also estimated among the four user groups using a Poisson regression model.

Analytical cross-sectional weights were used and the complex sampling design info (strata) were incorporated for all the analyses to make respondents within each of the user subgroups representative of the corresponding population with respect to demographics and region. All models were adjusted for sex, age group, income, and education. All analyses were conducted using the SAS-callable Sudaan v11 (Research Triangle Institute, Cary, NC, USA). All confidence intervals and statistical significance are tested at the 95% confidence level.

## 3. Results

### 3.1. Sample Characteristics

Most of the sample was male (70.1%), 40 years old or older (68.8%), and exclusive cigarette smokers (71.3%; see Table 1). 

### 3.2. Exposure to HTP Advertising by Channel

Across all user groups, exposure to HTP advertising was the highest on store windows or inside stores where tobacco and HTPs are sold, ranging from 30.5% of non-users to 69.6% of HTP users and 70.9% of dual users (see Table 2). Exposure to HTP advertising through posters/billboards ranged from about one in five non-users to one-third of smokers and dual users. Across all user groups, exposure was lowest in bars/pubs (ranging from 5.4% of HTP users to 10.9% of dual users) and on radio (2.8% of smokers to 8.4% of dual users).

Dual users and HTP users reported similar levels of exposure to HTP advertising across all channels, except for newspapers/magazines (28.6% vs. 18.5%, *p* < 0.05), bars and pubs (10.9% vs. 5.4%, *p* < 0.05), and radio (8.4% vs. 3.8%, *p* < 0.05). Smokers and HTP users reported similar levels of exposure across all channels except through email/text (10.5% vs. 28.1%, *p* < 0.001). Smokers reported higher exposure to HTP advertising than non-users across five channels: stores, posters/billboards, email/text messages (all comparisons *p* < 0.001), web/social media *(p* < 0.01), and newspapers/magazines (*p* < 0.05). HTP users and non-users reported the same level of exposure to HTP advertising on posters/billboards (25.7% vs. 20.0%), television (23.7% vs. 18.6%), and newspapers/magazines (18.5% vs. 19.0%).

Poisson regression models showed that males were more likely to be exposed to HTP advertising on television (*p* < 0.05), radio (*p* < 0.05), newspapers/magazines (*p* < 0.05), websites/social media (*p* < 0.01), and in bars and pubs (*p* < 0.05) compared to females. Younger respondents aged 20 to 29 years were more likely to be exposed to HTP advertising on websites/social media (*p* < 0.05) and less likely to be exposed to HTP advertising on radio (*p* < 0.001) compared to those aged 60 years and older. Respondents under age 40 years were more likely to be exposed to HTP advertising in bars/pubs (*p* < 0.05) compared to respondents aged 60 and older. Respondents under age 60 years were more likely to be exposed to HTP advertising on posters/billboards (*p* < 0.001) and on store windows or inside stores where HTPs are sold (*p* < 0.001) compared to those aged 60 years and older. Respondents aged 40 to 59 years were less likely to be exposed to HTP advertising on television (*p* < 0.05) compared to those aged 60 years and older. Respondents under 40 years old were more likely to be exposed to HTP advertising in bars or pubs (*p* < 0.001 and *p* < 0.05, respectively) compared to those aged 60 years and older. Respondents from high income groups were more likely to be exposed to HTP advertising in newspapers/magazines (*p* < 0.05), on posters/billboards (*p* < 0.001), and on shop windows/or inside stores where HTPs are sold (*p* < 0.001) compared to respondents from low income groups.

### 3.3. Exposure to Cigarette Advertising by Channel

Exposure to cigarette advertising was highest on store windows or inside stores where tobacco is sold across all user groups ranging from 43.4% of non-users to 66.0% of dual users (see Table 3). Exposure was relatively high on posters and billboards—reported by about a third of smokers and dual users and a quarter of HTP users and non-users. Exposure to television advertising ranged from 18.5% of HTP users to 26.6–30.0% of non-users, smokers and dual users. Email/text advertising exposure ranged from 5.1% of non-users to 14.5–21.6% among smokers, HTP users, and dual users. Exposure to cigarette advertising in bars/pubs (8.5% to 12.5%) and radio (3.5% to 6.5%) was relatively low across all groups. In contrast to HTP advertising, there were fewer channels (stores and email/text messages) where non-users reported lower exposure than smokers and dual users (*p* < 0.001), and HTP users (*p* < 0.01). Non-users reported the same levels of exposure to television and newspaper/magazine cigarette advertising (26.6% and 20.9%, respectively) as cigarette smokers (26.6% and 21.5%) and dual users (30.0% and 25.1%). Website/social media advertising exposure was higher among smokers and dual users (15.0% and 18.4%) compared to HTP users and non-users (9.4% and 10.0%, *p* < 0.05 for all comparisons except *p* < 0.01 for dual users vs. HTP users).

Poisson regression models showed that males were more likely to be exposed to cigarette advertising by email/text and in bars/pubs (both *p* < 0.01) compared to females. Respondents under 40 years old were more likely to be exposed to cigarette advertising in bars/pubs (*p* < 0.05) compared to respondents aged 60 years and older. Respondents aged 30 to 39 years were more likely to be exposed to cigarette advertising on posters/billboards (*p* < 0.01) and on store windows and inside stores where tobacco is sold (*p* < 0.05) compared to respondents aged 60 years and older. Respondents from high income groups were more likely to be exposed to cigarette advertising in newspapers/magazines (*p* < 0.01) and compared to respondents from low income groups. Respondents with high education were more likely to be exposed to cigarette advertising on websites and social media (*p* < 0.05) compared to respondents with low education.

### 3.4. Comparison of Overall Average Exposure to Cigarette and HTP Advertising

**Figure 1:**
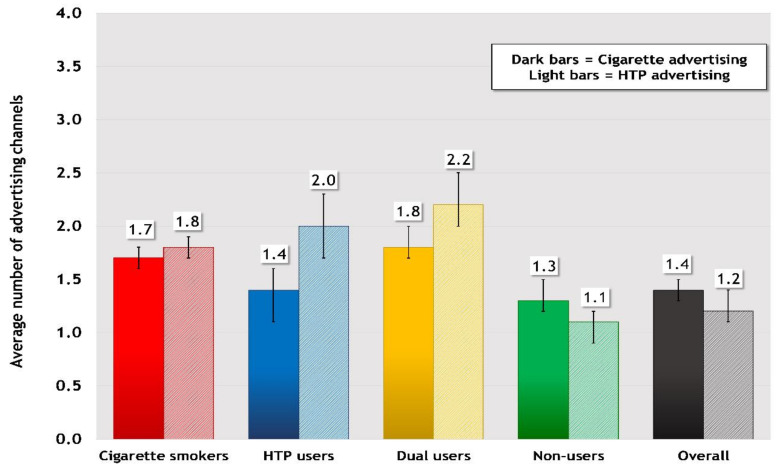
Average Number of Channels through which Cigarette and Heated Tobacco Product (HTP) Advertising was Noticed in the Last 6 Months †Respondents were asked whether they noticed cigarette and HTP advertising in 8 channels common to both forms of tobacco: TV, radio, newspapers or magazines, posters or billboards, in stores (for cigarette advertising: on store windows or inside stores where tobacco is sold; for HTP advertising: on store windows or inside stores where tobacco is sold + on store windows or inside stores where HTPs are sold), email, social media, and bars or pubs. Results are shown for the average number of channels for which respondents reported noticing advertising for each type of tobacco in the last 6 months. Overall refers to the entire sample of respondents.

Figure 1 presents the estimates of the average number of advertising channels to which respondents reported exposure in the last six months by user group and for the overall sample.

There was higher average exposure to cigarette advertising compared to HTP advertising among non-users (1.3 vs. 1.1 channels, *p* < 0.001) and the overall sample (1.4 vs. 1.2 channels, *p* < 0.01). However, average exposure to HTP advertising was higher than cigarette advertising for cigarette smokers (1.8 vs. 1.7 channels, *p* < 0.05), HTP users (2.0 vs. 1.4, *p* < 0.001), and dual users (2.2 vs. 1.8, *p* < 0.001).

Poisson regression models showed that tobacco users (*p* < 0.001), males (*p* < 0.01), younger age groups (*p* < 0.05), and high income groups (*p* < 0.001) were more likely to be exposed to the eight channels of HTP advertising than non-users, females, those aged 60 years and older, and low income groups. Cigarette smokers (*p* < 0.001), dual users (*p* < 0.001), males (*p* < 0.05) and high income groups (*p* < 0.01) were more likely to be exposed to the eight channels of cigarette advertising compared to non-users, females, and low income groups.

### 3.5. Support for HTP and Cigarette Marketing Bans

**Figure 2:**
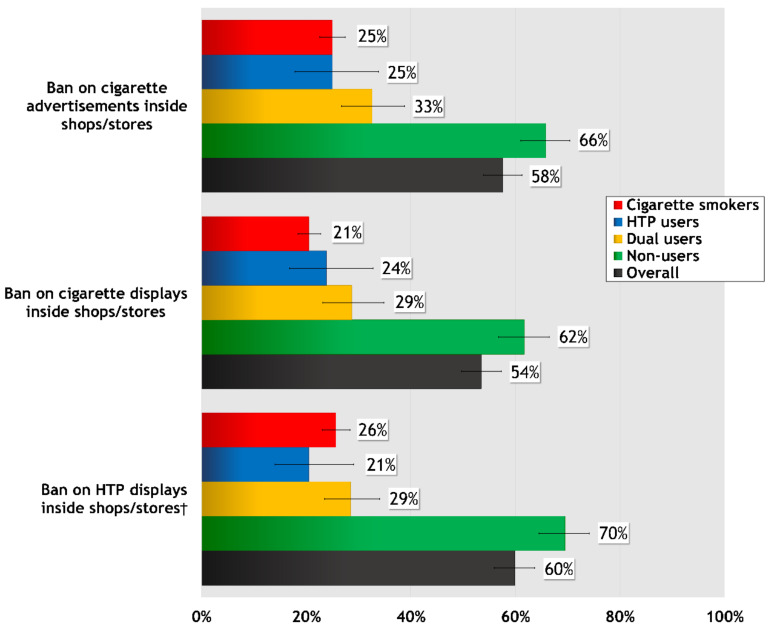
Percentage of Respondents who Support Tobacco Advertising, Promotion, and Sponsorship (TAPS) bans on HTPs and Cigarettes “Somewhat” or “A Lot”, Overall and by User Group. † This question was only asked to those who had ever heard of HTPs. Overall refers to the entire sample of respondents.

Figure 2 presents levels of support for: (a) a ban on cigarette displays inside shops/stores; (b) a ban on HTP displays inside shops/stores; and (c) a ban on all cigarette advertising inside shops and stores.

Overall, more than half of respondents supported cigarette (54%) and HTP (60%) display bans, and a ban on cigarette advertising in stores (58%). Support was highest among non-users for each policy measure, ranging from 61.7% for a cigarette display ban (*p* < 0.001 for all comparisons) to 69.5% for an HTP display ban (*p* < 0.001 for all comparisons)—at least double the level of support among smokers (20.5–25.7%), HTP users (20.5–25.0%), and dual users (28.5–32.6%). Compared to smokers, dual users reported higher support for cigarette advertising bans in stores (25.0% vs. 32.6%, *p* < 0.01) and for cigarette display bans (20.5% vs. 28.7%, *p* < 0.01). However, there were no differences in support for an HTP display ban among dual users, smokers, and HTP users (28.5%, 25.7%, and 20.5%, respectively).

## 4. Discussion

To our knowledge, this is the first population study in Japan to examine exposure to HTP and cigarette advertising. Respondents reported substantial exposure to HTP and cigarette advertising across a broad range of channels in 2018, including those that are highly visible to non-users and people of all ages. The most prevalent exposure to cigarette and HTP marketing was inside and outside stores, on store windows, posters and billboards, and on television and newspapers. Of concern are similar rates of reported exposure to cigarette and HTP advertising among product users and non-users through mass media channels. For example, there were no differences in the prevalence of cigarette advertising exposure reported by non-users through television (27%) and newspapers/magazines (21%) compared to smokers (27%, 22%) and dual users (30%, 25%). Similarly, there were no differences in reported exposure to HTP advertising by non-users through television (19%) and newspapers/magazines (19%) compared to HTP users (24% and 19%).

The data also show substantial exposure through more targeted advertising channels such as email/text messages and websites/social media which generally appeal to younger audiences and pose greater challenges for regulation. For example, survey respondents in the youngest age category (20–29 years) reported significantly higher exposure to HTP advertising through websites/social media compared to respondents aged 60 and older. In contrast, respondents aged 60 and older were more likely to be exposed to HTP advertising through traditional mass media channels such as television and radio compared to younger age groups. About a third of HTP users and dual users reported noticing HTP advertising through email/text messages (28% and 30%) and websites/social media (27% and 28%). In addition, about one in five cigarette smokers reported noticing HTP advertising on websites/social media (22%). Smokers and dual users reported lower exposure to cigarette advertising through these channels, however it was still substantial—15% of smokers and 18% of dual users for website/social media advertising and 16% of smokers and 22% of dual users for email/text message advertising. HTP users reported the same rate of exposure to cigarette advertising through email/text messages as smokers (15% vs. 16%).

High exposure to HTP and cigarette advertising are consistent with the absence of a comprehensive advertising ban on tobacco products in Japan and evidence from other countries on exposure to tobacco advertising in unregulated channels. Pervasive global HTP marketing through prominent displays in convenience stores, duty-free stores, dedicated IQOS boutique stores, Internet and social media channels such as Instagram, Facebook, and Twitter and a spectrum of other youth-oriented events are well described in recent reviews [13,40,41]. Between 2017 and 2019, PMI reported a three-fold increase in the number of stand-alone IQOS stores worldwide from 63 to 199 and more than twice the number of retailers selling HTPs from 292 to 679 [42]. Although PMI has suspended the widespread use of underage social media influencers to promote IQOS after findings of a Reuters investigation were released in May 2019 [43], a white paper on IQOS global marketing worldwide through to February 2020 notes that youth appealing marketing still continues [41].

The findings of this study demonstrate the consequences of Japan not meeting its obligations to Article 13 of the FCTC. Industry self-regulation, which calls for its own restrictions on tobacco advertising in “highly public areas” has clearly not prevented advertising to be noticed in those locations. The Tobacco Institute of Japan (TIOJ or Japan Tobacco Association) has adopted more restrictive voluntary industry standards for marketing of manufactured tobacco and heated tobacco products effective 1 July 2020 [44]. The new standards ban advertising on TV, radio, movies, public transportation and recorded media such as CDs or DVDs; require a text-only health warning in places or media where tobacco advertising is allowed; restrict the use of social influencers and youth to promote tobacco; and require age validation to prevent youth access to internet advertisements. However, the revised self-regulation does not prohibit advertising in the most widely visible channels (i.e., stores, posters/billboards, newspapers, and magazines). Therefore, these voluntary marketing restrictions fall short of meeting the best practice requirements of the WHO FCTC.

Evidence from the United Kingdom, Uruguay, Ireland, and Canada show dramatic reductions in exposure to tobacco marketing in channels after they are banned [45,46,47,48,49]. Studies indicate greater reductions in tobacco consumption following comprehensive marketing bans compared to bans in selective channels [50]. In addition, there is evidence that point of sale tobacco, advertising and display bans reduce adult smoking prevalence, tobacco normalization, and youth initiation [51,52,53,54].

Regulatory approaches for HTPs vary widely by country, however countries such as Canada and Israel have adopted strong regulations to curb HTP advertising, including a requirement for standardized packaging [55,56].

Without well-enforced comprehensive tobacco marketing restrictions, it can be expected that marketing of HTPs across multiple channels, including dedicated HTP stores, retail establishments, and e-commerce websites will intensify as JT and BAT compete for market share [16,57]. Findings of this paper demonstrate considerable support among tobacco users and non-users in Japan for policies to curb advertising of cigarettes and HTPs in retail settings, including a POS display ban for cigarettes and HTPs and a comprehensive ban on cigarette advertising in stores which were supported by a third of dual users and more than 60% of non-users.

This study has several limitations. First, this study relied on self-reported exposure to advertising in the last six months and therefore may be subject to recall bias. However, this measure is widely used in studies of exposure to marketing of tobacco and nicotine products. Second, the estimation of average exposure to cigarette and HTP marketing does not consider differences between advertising channels in the strength of influence of exposure on product uptake. Future studies could explore assigning different weights to various advertising channels and utilizing more precise measures of advertising exposure. Third, although nicotine-containing electronic cigarettes are not commercially available in Japan, some respondents may have been exposed to advertising of these products online and through social media. Thus, respondents may not have differentiated between advertising of electronic cigarettes and HTPs through these channels. Finally, the study sampling design and weights construction aim to ensure national representativeness, however the possibility of excluding participants in an online survey cannot be ruled out.

## 5. Conclusions

The absence of a comprehensive, strongly enforced ban on marketing of cigarettes and HTPs in Japan has resulted in pervasive public exposure to tobacco advertising. Regulators should fully implement a comprehensive marketing ban in line with the FCTC Article 13 to shift social norms and attitudes away from all forms of tobacco use and to strengthen Japan’s commitment to full implementation of the Treaty.

## Figures and Tables

**Table 1:** International Tobacco Control (ITC) Japan Wave 1 Survey Sample Characteristics.

Characteristic	Unweighted Frequency (%)
	(*n* = 4615)
Sex	
Female	1380 (29.9%)
Male	3235 (70.1%)
Age	
20–29	474 (10.3%)
30–39	963 (20.9%)
40–59	1940 (42.0%)
60+	1238 (26.8%)
Income	
Low	1212 (26.3%)
Moderate	1042 (22.6%)
High	1779 (38.5%)
No answer	582 (12.6%)
Education	
Low	1643 (35.6%)
Moderate	839 (18.2%)
High	2082 (45.1%)
No answer	51 (1.1%)
User group	
Exclusive HTP ^1^ users	164 (3.5%)
Exclusive cigarette smokers	3288 (71.3%)
Dual users of cigarettes and HTPs	549 (11.9%)
Non-users	614 (13.3%)

^1^ Heated Tobacco Products.

**Table 2:** Percentage of Respondents (and 95% CI) who Reported Noticing Advertising of HTPs in the Past Six Months by Channel and User Group.

Advertising Channel	Exclusive Cigarette Smoker (*n* = 3288)	Exclusive HTP User (*n* = 164)	Dual User (*n* = 549)	Non-User (*n* = 614)
On store windows or inside stores where tobacco or HTPs are sold	62.3 (59.3–65.2)	69.6 (58.9–78.6)	70.9 (64.5–76.6)	30.5 (26.0–35.3)
Posters/billboards	33.3 (30.4–36.3)	25.7 (18.7–34.1)	34.0 (28.6–39.8)	20.0 (16.1–24.6)
Email/text messages	10.5 (8.8–12.6)	28.1 (20.3–37.5)	30.2 (24.0–37.3)	3.9 (2.4–6.2)
Websites/social media	21.5 (18.8–24.4)	27.2 (19.4–36.8)	27.7 (22.3–33.9)	14.0 (10.3–18.8)
Television	23.2 (20.7–26.0)	23.7 (16.1–33.5)	29.1 (23.3–35.5)	18.6 (14.8–23.1)
Newspapers/magazines	24.9 (22.4–27.6)	18.5 (12.2–27.0)	28.6 (23.3–34.5)	19.0 (15.2–23.5)
Bars/pubs	8.4 (6.9–10.3)	5.4 (2.6–10.7)	10.9 (8.1–14.7)	6.6 (4.2–10.1)
Radio	2.8 (2.0–3.7)	3.8 (1.7–8.3)	8.4 (5.6–12.4)	3.5 (2.0–6.3)

**Table 3:** Percentage of Respondents (and 95% CI) who Reported Noticing Advertising of Cigarettes in the Past Six Months by Channel and User Group.

Advertising Channel	Exclusive Cigarette Smoker (*n* = 3288)	Exclusive HTP User (*n* = 164)	Dual User (*n* = 549)	Non-User (*n* = 614)
On store windows or inside stores where tobacco is sold	64.3 (61.4–67.0)	62.1 (51.4–71.9)	66.0 (59.8–71.7)	43.4 (38.2–48.6)
Posters/billboards	32.6 (29.9–35.4)	23.4 (16.5–32.3)	34.3 (28.8–40.3)	24.1 (19.9–28.9)
Television	26.6 (24.0–29.3)	18.5 (12.1–27.3)	30.0 (24.2–36.5)	26.6 (22.2–31.5)
Newspapers/magazines	21.5 (19.2–24.1)	14.0 (8.4–22.4)	25.1 (20.0–31.1)	20.9 (16.9–25.5)
Email/text messages	16.1 (14.2–18.3)	14.5 (9.3–22.0)	21.6 (16.7–27.5)	5.1 (3.2–8.0)
Websites/social media	15.0 (13.0–17.4)	9.4 (5.3–16.4)	18.4 (13.9–24.0)	10.0 (6.9–14.4)
Bars/pubs	10.5 (8.8–12.5)	8.5 (4.9–14.3)	11.0 (8.2–14.6)	12.5 (8.9–17.4)
Radio	3.5 (2.6–4.6)	3.6 (1.4–8.8)	6.5 (4.2–9.9)	4.5 (2.7–7.3)
